# Incidence and risk factors for symptomatic gallstone disease after gastrectomy for gastric cancer: a nationwide population-based study

**DOI:** 10.1097/JS9.0000000000004771

**Published:** 2026-01-19

**Authors:** Seohee Choi, Taemi Youk, Jawon Hwang, Sung Hyun Park, Minah Cho, Yoo Min Kim, Woo Jin Hyung, Hyoung-Il Kim

**Affiliations:** a Department of Surgery, National Health Insurance Service Ilsan Hospital, Goyang, Republic of Korea; b Research Institute, National Health Insurance Service Ilsan Hospital, Goyang, Republic of Korea; c Department of Surgery, Yonsei University College of Medicine, Seoul, Republic of Korea; d Gastric Cancer Center, Yonsei Cancer Center, Yonsei University Health System, Seoul, Republic of Korea

**Keywords:** cholecystectomy, cohort study, gallstones, gastrectomy, gastric cancer, laparoscopic surgery

## Abstract

**Background:**

Gallstone formation is a potential long-term complication of gastrectomies. However, data on symptomatic gallstone disease after gastrectomy for gastric cancer are limited. This nationwide population-based study aimed to determine the incidence and risk factors of symptomatic gallstone disease requiring invasive intervention.

**Method:**

This nationwide cohort study was based on claims data from the Korean National Health Insurance Service Database. The study included 90 456 patients who underwent gastrectomy for gastric cancer between 2007 and 2020 after excluding individuals with prior gallbladder disease, liver dysfunction, or ursodeoxycholic acid use. The primary outcome was symptomatic gallstone disease that required invasive intervention (cholecystectomy or endoscopic/percutaneous biliary procedures). Hazard ratios (HRs) and 95% confidence intervals (CIs) were estimated using the Cox proportional hazards analysis.

**Results:**

During a mean follow-up of 7.5 years, 6465 patients (7.1%) developed symptomatic gallstone disease requiring invasive intervention, with 5-year and 10-year cumulative incidences of 4.9% and 8.9%, respectively. Independent risk factors included age 60–79 years (HR 1.49, 95% CI 1.25–1.78) and ≥ 80 years (HR 2.10, 95% CI 1.69–2.61), body mass index ≥ 25 kg/m^2^ (HR 1.25, 95% CI 1.19–1.32), hypertension (HR 1.10, 95% CI 1.04–1.16), diabetes mellitus (HR 1.10, 95% CI 1.04–1.17), Charlson Comorbidity Index ≥ 6 (HR 1.32, 95% CI 1.23–1.43), total gastrectomy (HR 1.80, 95% CI 1.70–1.90), and adjuvant chemotherapy (HR 2.11, 95% CI 1.98–2.24). Female sex (HR 0.76, 95% CI 0.71–0.82), pylorus-preserving gastrectomy (HR 0.47, 95% CI 0.33–0.67), and laparoscopic surgery (HR 0.85, 95% CI 0.81–0.90) were protective.

**Conclusion:**

Symptomatic gallstone disease requiring invasive intervention occurred in 7.1% of the patients after gastrectomy for gastric cancer, representing a substantial increase compared to the general population. Pylorus-preserving gastrectomy and laparoscopic surgery were associated with a lower risk, suggesting that the surgical approach may influence the long-term gallstone risk.

## Introduction

Gastric cancer is the fifth most commonly diagnosed malignancy and the fifth leading cause of cancer-related deaths worldwide[1]. Gastrectomy is the standard curative treatment for gastric cancer, and advances in surgical techniques and perioperative management have significantly improved patient survival^[2,3]^. As postoperative survival continues to improve, attention has increasingly shifted toward managing long-term complications and enhancing quality of life after gastrectomy^[4-8]^.

Gallstone formation is a well-recognized long-term complication of gastrectomy^[9-11]^. Disruption of the gastrointestinal anatomy following gastrectomy, including nerve denervation and impaired bile flow, significantly increases the likelihood of gallbladder sludge and stone formation[12]. When symptomatic gallstone disease develops, surgical management can be technically demanding because postoperative adhesions increase the risk of bile duct injury, conversion to open surgery, and prolonged operative time, whereas endoscopic management is often hindered by altered gastrointestinal anatomy after gastrectomy^[13–15]^. Despite these important clinical implications, large-scale real-world data on the incidence and risk factors of symptomatic cholelithiasis after gastrectomy remain limited^[16,17]^. Unlike previous single-institution studies with limited patient numbers and short-term follow-up, our study utilizes a nationwide population-based with long-term follow-up, enabling a more comprehensive analysis. To provide clinically meaningful outcome measures, symptomatic gallstone disease was defined as case requiring invasive intervention rather than including asymptomatic disease detected on surveillance imaging.

This study aimed to investigate the incidence of symptomatic gallstone disease requiring invasive intervention after gastrectomy for gastric cancer using large-scale real-world data from the Korean National Health Insurance Service (NHIS). We also identified patient- and surgery-related risk factors associated with the development of symptomatic gallstone disease to guide preventive strategies and improve postoperative management in this patient population.

## Method

### Study population

**Figure 1.**
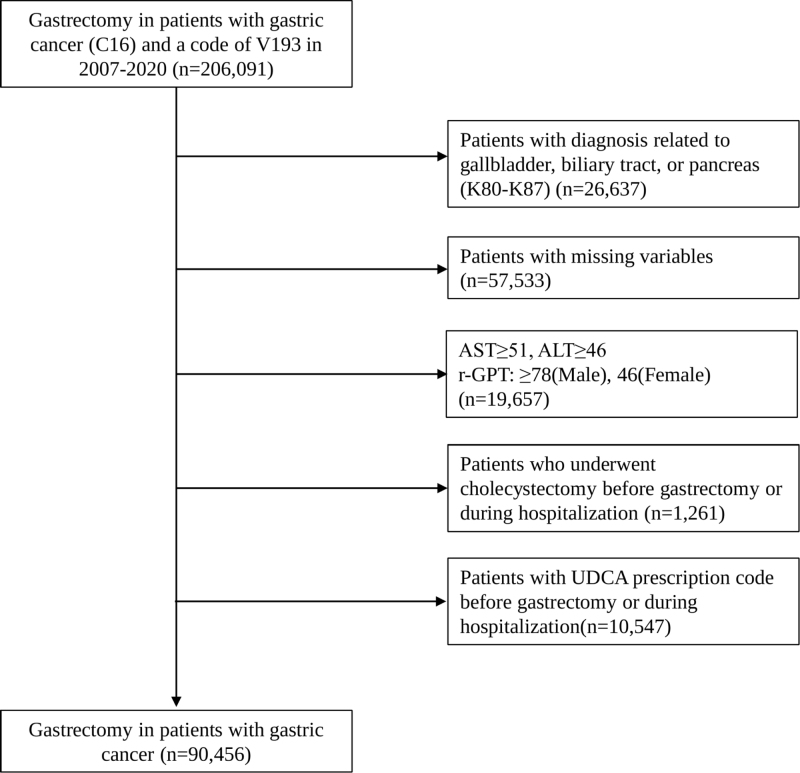
Flow chart of patient selection for the study cohort.

We initially identified 206 091 patients aged ≥19 years who underwent gastrectomy for gastric cancer between January 1, 2007 and December 31, 2020. Inclusion criteria: (1) adult patients aged 19 years or older; (2) underwent curative gastrectomy for primary gastric cancer (identified by ICD-10 code C16 and gastrectomy procedure codes) during the study period (2007–2020); (3) had available data from the National Health Screening Program within 2 years before surgery, including body mass index (BMI), laboratory results, and lifestyle factors (alcohol consumption and smoking status). Exclusion criteria: (1) patients with diagnoses related to gallbladder, biliary tract, or pancreas (ICD-10 codes K80–K87); (2) patients lacking required variables from the national health screening evaluation within 2 years before gastrectomy; (3) patients with suspected liver disease, defined as aspartate aminotransferase (AST) ≥ 51 U/L, alanine aminotransferase (ALT) ≥ 46 U/L, or gamma-glutamyl transferase (GGT) ≥ 78 U/L for men and ≥ 46 U/L for women; (4) patients prescribed ursodeoxycholic acid (UDCA) before or during hospitalization for gastrectomy; (5) patients who underwent cholecystectomy, cholecystostomy or common bile duct exploration procedures (procedure codes Q7380, Q7390, Q7310) or who underwent endoscopic/percutaneous biliary interventions (procedure codes Q7761–Q7767, Q7771-Q7776, M6670, M6681, M6682) before or during hospitalization for gastrectomy. After applying these criteria, 90 456 patients were included in the final analysis (Fig. 1). This cohort study has been reported in line with the STROCSS guidelines[18].

### Data source and variables

In this study, we used data from the Korean NHIS database, which provides claims data covering more than 98% of the Korean population, along with results from the national general health-screening program. Demographic characteristics (age, sex, income level), comorbidities, Charlson Comorbidity Index (CCI), surgical details, chemotherapy administration, hospitalization records, and mortality data were obtained from NHIS claims data. Clinical measurements such as height, weight, and BMI, as well as survey information on smoking, alcohol consumption, and laboratory test results (AST, ALT, and GGT), were extracted from the general health screening database. Only health screening results obtained within 2 years before gastrectomy were included. Comorbidities, such as hypertension, diabetes mellitus, dyslipidemia, liver disease, and prior gallbladder diseases, were identified using International Classification of Diseases, 10th Revision (ICD-10) codes. The CCI score was calculated based on the presence of 16 comorbid conditions identified within 2 years prior to gastrectomy. Gastrectomy type (distal, pylorus-preserving, proximal, or total gastrectomy) was classified based on the procedure codes, and the surgical approach (open versus laparoscopic) was determined by the presence or absence of a fixed-cost laparoscopic device code. Adjuvant chemotherapy was identified based on anticancer drug codes, in accordance with the guidelines of the Korean Gastric Cancer Association guidelines[19].

**Highlights**

- This nationwide cohort of 90 456 patients who underwent gastrectomy for gastric cancer showed a 7.1% incidence of symptomatic gallstone disease requiring intervention over a mean follow-up of 7.5 years.
- Total gastrectomy and adjuvant chemotherapy significantly increased the risk, whereas pylorus-preserving gastrectomy and laparoscopic surgery were associated with reduced risk.
- These findings suggest that individualized preventive strategies should be considered, and when surgically feasible, pylorus-preserving or laparoscopic approaches may help reduce the long-term gallstone risk.

### Outcome definition

The primary study outcome was the development of symptomatic gallstone disease after gastrectomy that required invasive intervention. Invasive intervention was defined as (1) cholecystectomy (procedure codes Q7380) or cholecystostomy (Q7390); (2) common bile duct exploration procedures, including choledochotomy or choledocholithotomy (Q7310); (3) endoscopic or percutaneous biliary procedures such as endoscopic retrograde cholangiopancreatography (ERCP; procedure codes Q7761–Q7767) and percutaneous transhepatic biliary drainage (PTBD; procedure codes Q7771–Q7776, M6670, M6681, M6682). All procedures were identified using the NHIS claims database.

### Statistical analysis

Categorical variables were summarized as frequencies and percentages and compared using the chi-square test. Continuous variables are expressed as means with standard deviations (SDs) and compared using an independent *t*-test. Risk factors for symptomatic gallstone disease requiring invasive intervention were assessed using Cox proportional hazards analysis and hazard ratios (HRs) with 95% confidence intervals (CIs) were calculated. Two-sided *P-*values < 0.05 were considered indicative of statistical significance. All statistical analyses were performed using the SAS software (version 9.4; SAS Institute Inc., Cary, NC, USA).

## Results

### Patient characteristics

**Figure 2.**
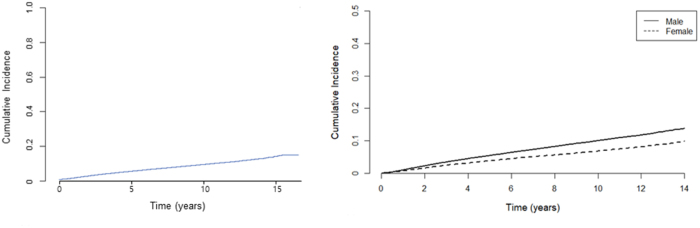
Cumulative incidence of symptomatic gallstone disease requiring invasive intervention after gastrectomy for gastric cancer. (A) Overall cumulative incidence; (B) cumulative incidence stratified by sex.

In total, 90 456 patients who underwent gastrectomy for gastric cancer were included in this study. The mean follow-up duration was 7.5 years (SD, 3.9 years). During follow-up, 21 169 patients (23.4%) died and 6465 patients (7.1%) developed symptomatic gallstone disease requiring invasive intervention. The cumulative incidence of symptomatic gallstone disease is shown in Figure 2. The incidence increased gradually over time (Fig. 2A) and remained consistently higher in males than in females throughout the follow-up period (Fig. 2B).

Table 1 summarizes patient characteristics according to the presence or absence of symptomatic gallstone disease requiring invasive intervention. Patients requiring invasive intervention for symptomatic gallstone disease were more likely to be male (invasive intervention vs. no invasive intervention: 71.4% vs. 63.7%, *P* < 0.001) and older (60–79 years: 60.2% vs. 52.4%; ≥ 80 years: 4.0% vs. 4.0%; *P* < 0.001) than those without invasive intervention. A BMI ≥ 25 kg/m^2^ (36.6% vs. 30.6%, *P* < 0.001), hypertension (45.1% vs. 39.3%, *P* < 0.001), and diabetes mellitus (30.4% vs. 25.9%, *P* < 0.001) were more common in the invasive intervention group. A higher proportion of patients in the invasive intervention group were current smokers (27.9% vs. 25.6%, *P* < 0.001), whereas alcohol use did not differ significantly between the groups (*P* = 0.121).

**Table 1:** Patient characteristics according to presence or absence of symptomatic gallstone disease requiring invasive intervention after gastrectomy for gastric cancer.

Variable	No invasive intervention		Invasive intervention		*P-*value
	*n* = 83 991	%	*n* = 6465	%	
Sex ratio (M: F)	53 476: 30 515	63.7: 36.3	4616: 1849	71.4: 28.6	<0.001
Age (years)					<0.001
19–39	2222	2.6	131	2.0	
40–59	34 419	41.0	2182	33.8	
60–79	43 996	52.4	3895	60.2	
≥ 80	3354	4.0	257	4.0	
Body mass index (kg/m^2^)					<0.001
< 25	58 298	69.4	4099	63.4	
≥ 25	25 693	30.6	2366	36.6	
Hypertension	33 023	39.3	2917	45.1	<0.001
Diabetes mellitus	21 788	25.9	1966	30.4	<0.001
Dyslipidaemia	29 812	35.5	2235	34.6	0.135
Liver disease	20 842	24.8	1700	26.3	0.008
Charlson Comorbidity Index					<0.001
2–3	31 269	37.2	2064	31.9	
4–5	32 849	39.1	2536	39.2	
≥ 6	19 873	23.7	1865	28.8	
Smoking					<0.001
Never	45 014	53.6	3191	49.4	
Former	17 483	20.8	1473	22.8	
Current	21 494	25.6	1801	27.9	
Alcohol use					0.121
No	47 552	56.6	3596	55.6	
Yes	36 439	43.4	2869	44.4	
Extent of gastrectomy					<0.001
Distal gastrectomy	62 961	75.0	4157	64.3	
Pylorus-preserving gastrectomy	1219	1.5	31	0.5	
Proximal gastrectomy	1534	1.8	99	1.5	
Total gastrectomy	18 277	21.8	2178	33.7	
Operative method					<0.001
Open	41 399	49.3	3934	60.9	
Laparoscopy	42 592	50.7	2531	39.1	
Adjuvant chemotherapy	14 518	17.3	1548	23.9	< 0.001

Total gastrectomy (33.7% vs. 21.8%, *P* < 0.001) and open surgery (60.9% vs. 49.3%, *P* < 0.001) were more frequently performed in patients requiring invasive intervention for symptomatic gallstone disease than in those not requiring invasive intervention. Furthermore, a higher proportion of patients in the invasive intervention group received adjuvant chemotherapy (23.9% vs. 17.3%, *P* < 0.001).

### Risk factors for symptomatic gallstone disease requiring invasive intervention

Table 2 shows the results of the multivariate analysis of potential risk factors for symptomatic gallstone disease. Compared with the male sex, the female sex was associated with a significantly lower risk (HR 0.76, 95% CI 0.71–0.82, *P* < 0.001). The risk of symptomatic gallstone disease requiring invasive intervention was higher in the 60–79 years (HR 1.49, 95% CI 1.25–1.78, *P* < 0.001) and ≥ 80 years (HR 2.10, 95% CI 1.69–2.61, *P* < 0.001) age groups than in the 19–39 years age group. BMI ≥ 25 kg/m^2^ was associated with an increased risk of requiring invasive intervention (HR 1.25, 95% CI 1.19–1.32; *P* < 0.001). CCI 4–5 (HR 1.11, 95% CI 1.04–1.18, *P* = 0.002), CCI ≥ 6 (HR 1.32, 95% CI 1.23–1.43, *P* < 0.001), hypertension (HR 1.10, 95% CI 1.04–1.16, *P* = 0.001), and diabetes mellitus (HR 1.10, 95% CI 1.04–1.17, *P* = 0.002) were also associated with an increased risk. Dyslipidemia, liver disease, former smoking, current smoking, and alcohol use were not significantly associated with symptomatic gallstone disease that required invasive intervention.

**Table 2:** Multivariable analysis of potential risk factors for symptomatic gallstone disease requiring invasive intervention after gastrectomy for gastric cancer.

Variable	Hazard Ratio	95% CI		*P-*value
		Lower	Upper	
Sex				
Male	1.00 (ref)			
Female	0.76	0.71	0.82	<0.001
Age (years)				
19–39	1.00 (ref)			
40–59	1.05	0.88	1.25	0.599
60–79	1.49	1.25	1.78	<0.001
≥ 80	2.10	1.69	2.61	<0.001
Body mass index (kg/m^2^)				
< 25	1.00 (ref)			
≥ 25	1.25	1.19	1.32	< 0.001
Hypertension	1.10	1.04	1.16	0.001
Diabetes mellitus	1.10	1.04	1.17	0.002
Dyslipidaemia	0.95	0.90	1.01	0.081
Liver disease	1.01	0.95	1.07	0.712
Charlson Comorbidity Index				
2–3	1.00 (ref)			
4–5	1.11	1.04	1.18	0.002
≥ 6	1.32	1.23	1.43	<0.001
Smoking				
Never	1.00 (ref)			
Former	1.02	0.94	1.09	0.689
Current	1.07	1.00	1.15	0.058
Alcohol use				
No	1.00 (ref)			
Yes	0.99	0.93	1.04	0.599
Extent of gastrectomy				
Distal gastrectomy	1.00 (ref)			
Pylorus-preserving gastrectomy	0.47	0.33	0.67	<0.001
Proximal gastrectomy	1.07	0.88	1.31	0.491
Total gastrectomy	1.80	1.70	1.90	<0.001
Operative method				
Open	1.00 (ref)			
Laparoscopy	0.85	0.81	0.90	<0.001
Adjuvant chemotherapy	2.11	1.98	2.24	<0.001

CI, confidence interval; HR, hazard ratio; ref, reference.

Compared with distal gastrectomy, pylorus-preserving gastrectomy (PPG) was associated with a lower risk of symptomatic gallstone disease requiring invasive intervention (HR 0.47, 95% CI 0.33–0.67; *P* < 0.001), whereas total gastrectomy was associated with a higher risk (HR 1.80, 95% CI 1.70–1.90; *P* < 0.001). Proximal gastrectomy was not associated with gallstone disease requiring invasive intervention. Laparoscopic surgery was associated with a lower risk than open surgery (HR 0.85, 95% CI 0.81–0.90; *P* < 0.001) and adjuvant chemotherapy was associated with a markedly higher risk than no adjuvant chemotherapy (HR 2.11, 95% CI 1.98–2.24; *P* < 0.001). Model-based estimates of the 5-year cumulative incidence across combinations of major risk factors were derived from the multivariable Cox proportional hazards model and are presented in Supplemental Digital Content Table 1,available at:  http://links.lww.com/JS9/G766.

## Discussion

In this large-scale nationwide cohort study of 90 456, patients who underwent gastrectomy for gastric cancer, 7.1% developed symptomatic gallstone disease requiring invasive intervention during a mean follow-up duration of 7.5 years. This incidence was substantially higher than rates previously reported in the general population. Multivariable analysis identified several independent risk factors for symptomatic gallstone disease requiring invasive intervention, including male sex, older age, higher BMI, hypertension, diabetes mellitus, and higher CCI. The risk was also significantly higher in patients who underwent total gastrectomy or received adjuvant chemotherapy. In contrast, laparoscopic surgery and PPG were associated with a lower likelihood of symptomatic disease requiring intervention.

The incidence of symptomatic gallstone disease requiring invasive interventions is higher in patients who undergo gastrectomy for gastric cancer than in the general population. According to statistics from the Korean NHIS, approximately 0.3% of the general population receives invasive treatment for gallstone disease annually, a rate similar to that reported in the United States. Previous studies have reported overall gallstone disease incidence rates ranging from 7.6% to 15% after gastrectomy, as these estimates generally included both symptomatic and asymptomatic cases^[20–22]^. When limited to symptomatic cases requiring intervention, the reported incidence ranges from 2.0% to 4.6%^[21,22]^, which is notably lower than the rate observed in the current study. This discrepancy may be attributed to differences in study design, including case definitions, methods of outcome ascertainment, and duration of follow-up. In particular, the extended observation period in our nationwide cohort and the inclusion of both surgical and nonsurgical biliary interventions may have contributed to the higher incidence rate.

Several patient-related characteristics were associated with an increased risk of symptomatic gallstone disease after gastrectomy. Older age has been linked to reduced physical activity and impaired gallbladder contractility, both of which promote bile stasis[23]. A high BMI increases hepatic cholesterol synthesis and biliary cholesterol secretion, contributing to cholesterol supersaturation, and has also been linked to impaired gallbladder motility after gastrectomy[24]. Diabetes mellitus may also impair gallbladder motility through autonomic neuropathy and metabolic dysfunction, leading to stasis and altered bile composition[25]. These mechanisms are similar to those described in the general population. Notably, although female sex is a well-established risk factor for gallstone disease in the general population, our findings indicated a higher risk among male patients[26]. This discrepancy may reflect the higher prevalence of gastric cancer and subsequent gastrectomy among men^[24,27]^.

Adjuvant chemotherapy was also identified as an independent risk factor for gallstone formation after gastrectomy. Although prior studies have reported inconsistent findings, a recent multicenter analysis suggested that chemotherapy may alter intestinal microbiota and bile composition, impairing gallbladder motility and promoting biliary stasis^[24,28,29]^. In addition, patients who receive adjuvant chemotherapy typically have more advanced gastric cancer requiring D2 lymph node dissection, including dissection along the hepatoduodenal ligament (No. 12 lymph node). This may lead to greater autonomic nerve injury and impaired gallbladder contraction independent of chemotherapy effects[30]. Clinically, these findings indicate that high-risk patients should be followed closely after gastrectomy to enable early detection and management of gallstone disease.

Beyond patient characteristics, several surgery-related factors also contribute to gallstone formation after gastrectomy. Total gastrectomy has been consistently associated with a higher risk of gallstone formation, likely due to transection of vagal trunk and dissection around the hepatoduodenal ligament, which impair gallbladder motility^[22,24,31]^. In addition, Roux-en-Y reconstruction commonly used after total gastrectomy bypasses the duodenum, the main site of cholecystokinin (CCK) secretion in response to fat ingestion. This anatomical alteration reduces CCK-mediated gallbladder contraction and promotes bile stasis[27]. Rapid postoperative weight loss further contributes to cholesterol supersaturation and gallstone formation, particularly in patients undergoing total gastrectomy who tend to experience more severe and sustained weight loss^[32,33]^. By contrast, certain surgical techniques and preventive strategies may reduce the likelihood of gallstone formation after gastrectomy. PPG, which is typically performed for early gastric cancer, is a type of gastrectomy associated with the lowest risk of gallstone development^[34,35]^. By maintaining vagal innervation and duodenal continuity, PPG preserves the neurohormonal signalling required for physiological gastric emptying and gallbladder contraction^[12,36]^. Laparoscopic gastrectomy was also associated with a significantly lower risk of symptomatic disease requiring invasive intervention, potentially by minimizing peritoneal adhesions and preserving physiological bile flow dynamics through less invasive manipulation[22]. Previous reports have also suggested that enhanced visualization provided by laparoscopic approaches enables better preservation of the hepatic branch of the vagus nerve[24]. Additionally, reduced postoperative ileus with earlier initiation of enteral feeding after laparoscopic procedures may help to prevent bile stasis, a key factor in gallstone formation[37]. Our findings suggest that when oncologically appropriate, PPG and laparoscopic surgery may not only confer functional benefits but also lower the long-term risk of gallstone-related complications, highlighting the need for further stage-stratified analyses to validate these protective associations.

Given the multifactorial etiology and clinical significance of gallstone disease following gastrectomy, preventive interventions have been investigated in randomized trials, most notably the CHOLEGAS trial evaluating prophylactic cholecystectomy and the PEGASUS-D trial assessing UDCA administration^[21,38]^. Prophylactic cholecystectomy performed at the time of gastrectomy has been shown to reduce the incidence of gallstones; however, long-term outcome data remain inconclusive^[39,40]^. A recent randomized controlled trial found that prophylactic cholecystectomy reduced the absolute number of biliary abnormalities, but did not significantly alter the natural course of the disease[21]. Similarly, postoperative UDCA has demonstrated efficacy in reducing gallstone formation in randomized trials[38]. In the Korean Gastric Cancer Association clinical practice guidelines, 1 year of UDCA administration after gastrectomy is conditionally recommended based on moderate-quality evidence[19]. These findings support the potential role of UDCA as a preventive option in selected patients; however, our study did not evaluate the effects of UDCA or prophylactic cholecystectomy. Therefore, these considerations should be interpreted as summaries of prior literature rather than conclusions derived from our analysis. Further studies are needed to identify patient subgroups who may benefit most from such preventive strategies and to clarify their long-term clinical impact.

This study has several limitations. First, our analyses were based on administrative claims data, which may have been subject to coding inaccuracies. These data also did not include detailed clinical information, such as extent of lymph node dissection, reconstruction, preservation of vagus nerve, nutritional and dietary factors, gallbladder imaging findings or symptom severity. Consequently, symptomatic gallstone disease was defined operationally as the receipt of invasive intervention. This definition may not capture all clinically relevant cases and could potentially include procedures performed for diagnostic purposes. However, this operational definition implicitly reflects cases of clinically meaningful severity, as it includes only patients who required therapeutic procedures such as cholecystectomy or biliary intervention. Second, although multivariable analyses were conducted to adjust for known confounders, causal inferences remain limited given the observational nature of the study. Although adjuvant chemotherapy was adjusted as a proxy for disease stage, residual confounding due to unmeasured cancer severity may still remain because information on pathologic TNM stage was not available in the claims data. Nevertheless, the nationwide claims database ensured comprehensive follow-up of gallstone-related interventions after gastrectomy across different healthcare providers, thereby minimizing the risk of outcome misclassification and enhancing the robustness of the results. Future prospective studies employing standardized diagnostic criteria and symptom-based definitions are required to validate our findings and to allow for more precise risk stratification.

In conclusion, this large-scale nationwide cohort study demonstrated a substantially increased incidence of symptomatic gallstone disease requiring invasive intervention after gastrectomy for gastric cancer. Risk factors independently associated with symptomatic disease requiring invasive intervention included total gastrectomy, adjuvant chemotherapy, and comorbidities such as diabetes and hypertension. These findings highlight the need for individualized risk assessment and suggest that PPG and laparoscopic surgery may benefit selected high-risk patients, when oncologically appropriate. Further prospective studies with standardized criteria and long-term follow-up are warranted to validate our findings and to guide preventive strategies.

## Data Availability

This study used NHIS-NSC data (NHIS-2023-1-666) from the National Health Insurance Service (NHIS) under licence for the current study. These data are not publicly available.

## Research registration unique identifying number (UIN)

Not applicable. This study used anonymized secondary data from a national claims database and did not involve prospective recruitment of human subjects.
